# Supplementary material for: MLH1 Promoter Variant −93G>A and Breast Cancer Susceptibility: Evidence from Azerbaijan
Source: Biomedicines. 2025 Nov 12;13(11):2769. doi: 10.3390/biomedicines13112769 (PMC12650484; doi:10.3390/biomedicines13112769)
Supplement: Supplementary file 1 [file biomedicines-13-02769-s001.zip › biomedicines-3957688-supplementary.pdf]

biomedicines

Supplementary materials:

Table S1. Clinical and pathological characteristics of study groups (expanded).

Parameter	Category	*n* (%)	Comment
**TNM classification**			
**T – Primary tumor**	T1	12 (8.4 %)	≤ 2 cm
	T2	65 (45.5 %)	2–5 cm
	T3	36 (25.1 %)	> 5 cm
	T4	30 (21.0 %)	Chest wall/skin invasion
**N – Regional lymph nodes**	N0	42 (29.4 %)	Node-negative
	N1	58 (40.6 %)	1–3 positive nodes
	N2	29 (20.2 %)	4–9 positive nodes
	N3	14 (9.8 %)	≥ 10 positive nodes
**M – Distant metastasis**	M0	87 (60.8 %)	No metastasis
	M1	56 (39.2 %)	Distant metastasis present
**CA 15-3 (U/mL)**	Mean ± SD (range)	78.4 ± 52.6 (26 – 300)	Elevated > 30 U/mL in ≈ 70 % of patients
	< 30 U/mL	43 (30.1 %)	Within reference
	≥ 30 U/mL	100 (69.9 %)	Elevated
**BMI (kg/m²)**	Mean ± SD (range)	28.1 ± 4.9 (19.0 – 39.5)	Calculated for all patients
**BMI category (WHO)**	< 18.5 – Underweight	2 (1.4 %)	
	18.5–24.9 – Normal	36 (25.1 %)	
	25.0–29.9 – Overweight	54 (37.8 %)	
	≥ 30.0 – Obese	51 (35.7 %)	

Table S2. Histological type and hormone-receptor status distribution among patients.

Histological Type	*n* (%)	ER+ / PR+ (%)	HER2+ (%)	Triple-Negative (%)
**Invasive ductal carcinoma (IDC)**	122 (85.3 %)	77 (63.1 %)	27 (22.1 %)	7 (5.7 %)
**Invasive lobular carcinoma (ILC)**	18 (12.6 %)	17 (94.4 %)	1 (5.6 %)	0 (0 %)
**Other special types**	3 (2.1 %)	3 (100 %)	0 (0 %)	0 (0 %)
**Total**	**143 (100 %)**	**97 (67.8 %)**	**28 (19.5 %)**	**7 (4.9 %)**

Table S3. Distribution of MLH1 −93G>A genotypes by histological type among patients.

Histological Type	*n*	GG (*n*, %)	GA (*n*, %)	AA (*n*, %)	*p* Value
**Invasive ductal carcinoma (IDC)**	122	32 (26.2 %)	71 (58.2 %)	19 (15.6 %)	0.76
**Invasive lobular carcinoma (ILC)**	18	4 (22.2 %)	11 (61.1 %)	3 (16.7 %)	
**Other special types**	3	1 (33.3 %)	2 (66.7 %)	0 (0 %)	
**Total**	**143**	**37 (25.9 %)**	**84 (58.7 %)**	**22 (15.4 %)**	
